# Optimization of the culture condition of *Bacillus mucilaginous* using *Agaricus bisporus* industrial wastewater by Plackett–Burman combined with Box–Behnken response surface method

**DOI:** 10.1186/s13568-018-0671-7

**Published:** 2018-08-31

**Authors:** Jiafu Huang, Yixin Ou, Danfeng Zhang, Guoguang Zhang, Yutian Pan

**Affiliations:** Engineering Technological Center of Mushroom Industry, Minnan Normal University, No. 36, Qianzhi Street, Zhangzhou, 363000 Fujian People’s Republic of China

**Keywords:** *Agaricus bisporus* wastewater, *Bacillus mucilaginous*, Multispectral imaging flow cytometry, Plackett–Burman, Box–Behnken response surface

## Abstract

In the present study, conditions for *Bacillus mucilaginous* fermentation using *Agaricus bisporus* wastewater as culture medium were optimized. We analyzed the total number of living *B. mucilaginous* in the fermentation broth using multispectral imaging flow cytometry. Single-factor experiments were carried out, where a Plackett–Burman design was used to screen out three factors from the original six factors of processing wastewater solubility, initial pH, inoculum size, liquid volume, culture temperature, and rotation speed that affected the total number of viable *B. mucilaginous*. The Box–Behnken response surface method was used to optimize interactions between the three main factors and predict optimal fermentation conditions. Factors significantly affecting the total number of viable *B. mucilaginous*, including shaking speed, culturing temperature, and initial pH, were investigated. The optimum conditions for *B. mucilaginous* fermentation in *A. bisporus* wastewater were a rotational speed of 195 rpm, culture temperature of 29 °C, initial pH of 6.5, solubility of 0.5%, 8% inoculation volume, and 90 mL liquid volume in a 250 mL flask, culture time of 48 h. Under these conditions, the concentration of total viable bacteria reached 2.16 ± 0.02 × 10^8^ Obj/mL, which meets the national standard. *A. bisporus* wastewater can be used for the cultivation of *B. mucilaginous*.

## Introduction

*Agaricus bisporus (Lange) Sing* is the most widely cultivated and most consumed edible mushroom with the highest yield in the world, accounting for about 25% of the total world edible fungus production. Due to the short storage period of fresh *A. bisporus*, the main form of international trade is mainly canned processed products. During tank processing, fresh mushrooms are promptly pre-cooked to prevent mushrooms umbrella, and the weight of cooked mushrooms is 35–40% lower than that of fresh mushrooms; the loss of weight goes to industrial wastewater—*A. bisporus* wastewater. In China, *A. bisporus* used for canned processing accounts for about 80% of the total production of *A. bisporus*. About 30% of *A. bisporus* production is lost to industrial wastewater every year. The mushroom pre-cooking liquid is a production wastewater with high BOD and COD content, of which the content of COD is 540.29 g/L, which is 13.07 times higher than the national three level emission standard. The direct discharge of pre-cooking liquid will cause environmental pollution, and wastewater treatment will increase the production cost (Huang et al. 2016); however, the water soluble nutrients can be extracted by pre-cooking fresh *A. bisporus*. The preboiled liquid of *A. bisporus* contains a variety of nutrients such as free protein, polysaccharides, mannitol, and mineral ions (Lin et al. 2016), so it can be used as a natural medium for the cultivation of certain beneficial bacteria or plants (Zhan et al. 2017). Therefore, the comprehensive utilization of this industrial wastewater is of practical significance for protecting the ecological system of origin and enhancing the comprehensive utilization of agricultural resources.

Since *B. mucilaginous* can transfer the insoluble phosphorus that is present as either an inorganic mineral such as apatite or as one of several organic forms including inositol phosphate (soil phytate), phosphomonesters, and phosphotriesters in the soil to support plant growth, fix nitrogen and provide it to plants (Rojas et al. 2001; Javadi Nobandegani et al. 2015; Kuan et al. 2016), and it also generates organic acids, amino acids, polysaccharides, hormones, and other substances absorbed and utilized by plants during growth and reproduction (Glick 2012; Koroney et al. 2016; Khalid et al. 2017; Schütz et al. 2017).

If the industrial wastewater produced in the industry of *A. bisporus* could be used as a natural medium for *B. mucilaginous* that is one of the most important functional bacteria in microbial fertilizers widely used in the agricultural industry, which would provide theoretical support for microbial fertilizers’ fermentation and the development of the downstream industry of *A. bisporus*.

## Materials and methods

### Strains, media, growth conditions and Instrumentation

*Bacillus mucilaginous* (GIM1.16) was purchased from the Guangdong culture collection center.

Inclined medium consisted of 20 g mannitol, 0.2 g KH_2_PO_4_, 0.8 g K_2_HPO_4_, 0.2 g MgSO_4_·7H_2_0, 0.1 g CaSO_4_·2H_2_0, trace Na_2_MoO_4_·2H_2_O, trace FeCl_3_, 0.5 g yeast extract, 15 g agar, and 1000 mL distilled water (pH 7.0–7.2) that had been sterilized for 15 min at 121 °C using wet heat.

Seed medium consisted of 20 g mannitol, 0.2 g KH_2_PO_4_, 0.8 g K_2_HPO_4_, 0.2 g MgSO_4_·7H_2_0, 0.1 g CaSO_4_·2H_2_0, trace Na_2_MoO_4_·2H_2_O, trace FeCl_3_, 0.5 g yeast extract, and distilled water 1000 mL (pH 7.0–7.2) that had been sterilized for 15 min at 121 °C using wet heat.

*Agaricus bisporus* processing wastewater was collected from the processing enterprises (Fujian KEREN Biological Co., Ltd.), filtered, and concentrated according to different experimental requirements. Deionized water was used for different solubilities and packaging. Sterilization was performed at 121 °C for 15 min.

Instrumentation used in this study included a multispectral imaging flow cytometer (FlowSight, Millipore, USA), biosafety cabinet (HFsafe-1500, Li Kang, Hong Kong), high-speed centrifuge (5810R, Eppendorf, Germany), autoclave (SE-50, Tomy, Japan), constant temperature incubator (760R, Shellab, USA), and pipette (Xplorer, Eppendorf, Germany).

### Strain activation

Lyophilized bacteria were activated for the first time by dissolving in 0.1–0.2 mL sterile water and inoculating onto two angular surfaces, which were placed at 28 °C for 24–48 h.

### Preparation of seed suspension

Activated *B. mucilaginous* was picked with a vaccination loop and transferred to a 100 mL flask containing sterilized seed liquid medium. This bacterial suspension was placed in a 28 °C incubator with shaking at 150 rpm for 48 h.

### *B. mucilaginous* growth curve

Every 2 h, 1 mL of bacterial suspension was aseptically removed from a shake flask culture and serially diluted 10-fold in PBS. One mL of the diluted solution and 3 μL LIVE/DEAD Baclight™ staining reagent were mixed and then incubated for 30 min in the dark. The total number of viable *B. mucilaginous* was then measured by flow cytometry. The culture duration and the total number of viable *B. mucilaginous* were considered the abscissa and ordinate, respectively, and the growth curve was generated (Dalton and Postgate 1969; Calvert Meredith et al. 2008).

### Single-factor test

In the single factor test, a fixed culture time of 48 h was used, and the remaining factors and levels were selected as described (Zhang et al. 2013; Kang et al. 2014; Ren et al. 2014) in Table 1. The single factor experiments were carried out and were repeated in triplicate. The total number of living *B. mucilaginous* in the fermentation broth was analyzed using multispectral imaging flow cytometry.

**Table 1:** Factors and levels assessed in single-factor tests

Level	Factor					
	Concentration (%)	pH	Loaded liquid (mL/250 mL)	Inoculum (*v:v*, %)	Culture temperature (°C)	Shaking speed (rpm)
1	0.0625	5.0	15	0.5	20	50
2	0.125	5.5	30	1	24	100
3	0.25	6.0	60	2	28	150
4	0.5	6.5	90	4	32	200
5	1	7.0	120	8	36	250
6	2	7.5	150	16	–	–
7		8.0	–	–	–	–

### Three main factors affecting total number of viable *B. mucilaginous* identified using Plackett–Burman design

Plackett–Burman is a near-saturated 2-level experimental design method based on the principle of partial complete equilibrium, which can estimate the effect of a factor using the minimum number of experiments and quickly and effectively identify the most important factors from many for further study. In this study, the influence of 6 culture conditions on the total number of viable *B. mucilaginous* was investigated. The experimental design included 6 factors and the experimental number selected was N = 12. A, B, C, D, E, and F represents precooked liquid solubility, initial pH, inoculum size, culture temperature, shaking speed, and liquid volume, respectively (Chen et al. 2015a, 2017a; Gao et al. 2016). Based on the results of the single-factor experiments, each factor was taken to two levels (Table 2).

**Table 2:** Plackett–Burman experimental design

Code run	A [concentration (%)]	B (pH)	C [inoculum (%)]	D [loaded liquid (mL/250 mL)]	E (culture temperature (°C))	F [shaking speed (rpm)]	Total viable *B. mucilaginous* (10^7^ Obj/mL)
1	1	− 1	1	1	1	− 1	1.15 ± 0.02
2	1	− 1	− 1	− 1	1	− 1	0
3	−1	1	1	1	− 1	− 1	5.30 ± 0.01
4	−1	1	1	− 1	1	1	1.78 ± 0.01
5	−1	− 1	1	− 1	1	1	3.75 ± 0.02
6	1	1	1	− 1	− 1	− 1	2.90 ± 0.01
7	−1	1	− 1	1	1	− 1	2.20 ± 0.01
8	−1	− 1	− 1	1	− 1	1	5.05 ± 0.03
9	1	1	− 1	− 1	− 1	1	5.10 ± 0.02
10	−1	− 1	− 1	− 1	− 1	− 1	1.25 ± 0.02
11	1	1	− 1	1	1	1	3.50 ± 0.03
12	1	− 1	1	1	− 1	1	4.17 ± 0.02

### Steepest ascent design

The response surface fitting equation needs to mimic the real situation in the immediate neighborhood of the investigation. Therefore, an effective response surface fitting equation must be established before the maximum response value area can be determined. The steepest ascent method changes the gradient direction of the experimental value into the direction of hill climbing. Based on the effect of each factor, the change step size can be determined, which can be used to quickly and economically approximate the optimal value area (Chen et al. 2015a; Gao et al. 2016). According to the size of the key factor effect value in the Plackett–Burman experiment, the change distance and direction of climbing can be determined. This can be used to determine the best level range.

### Box-Benhnken design

According to the results of the Plackett–Burman and steepest ascent experiments, the factors and levels for the Box–Benhnken design experiments were determined. To evaluate the impact of various factors on the total number of viable bacteria and identify the optimal fermentation conditions, three-factor three-level experimental design was utilized (Cui and Zhao 2012; Wang et al. 2016). In this design, the total number of viable cells was considered as the response value, the key three variables as the independent variables, and experimental design and data analysis were performed for each single factor using Design-Expert 8.0 software.

### Verification

To verify the reliability of the experimental model, three parallel experiments were performed according to the optimal fermentation conditions identified through Box–Benhnken design experiments. The resulting values were averaged to obtain the final results.

### Quantification of the total number of living bacteria

As described by Calvert et al. (2008), the fermentation broth was diluted 10-fold with PBS and 1 mL of the diluted solution was mixed and incubated with 3 μL LIVE/DEAD Baclight™ staining reagent in the dark for 30 min. The number of viable bacteria was then quantified by flow cytometry. PBS was used as a flow sheath and a 480 nm laser was used to collect fluorescence signals and images from the 20,000 bright field, channel 2 green fluorescence representing SYTO 9, and channel 5 red fluorescence representing propidium iodide (PI). The SYTO 9 signal was set as the X axis and the PI signal as the Y axis and a scatter plot was generated to distinguish between the dead and living bacteria.

## Results

### Growth curve

**Fig. 1:**
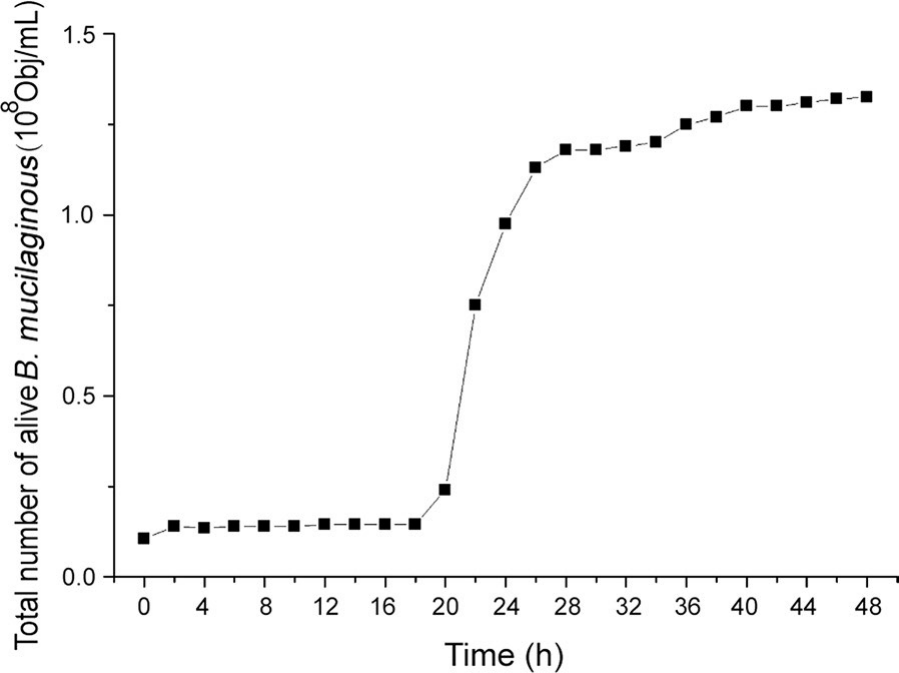
*B. mucilaginous* growth curve

As shown in Fig. 1, the growth curve of *B. mucilaginous* was a typical “S” curve. A lag period occurred from 0 to 20 h and was followed by a logarithmic growth period from 20 to 30 h, where the number of live bacteria increased rapidly and the maximum growth rate was reached. Following this period, the curve was stable from 32 to 48 h, which indicates the optimal period of fermentation. If culture duration was extended, bacterial competition for survival and a resulting decline in individual viable bacteria occurred.

### Detection of the total number of viable bacteria during fermentation

**Fig. 2:**
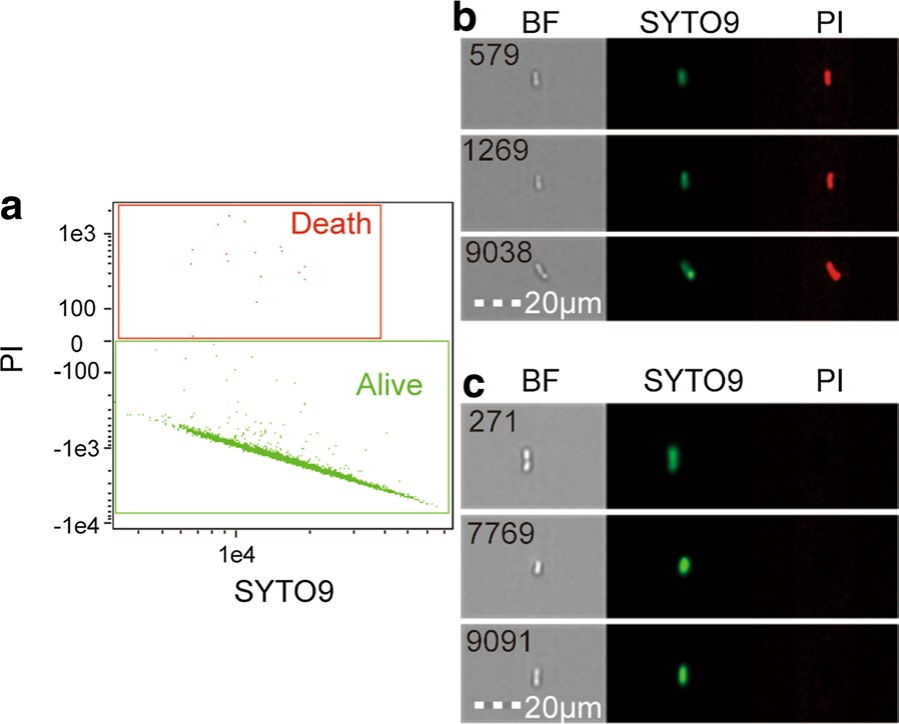
Live and dead *B. mucilaginous* based on multispectral imaging flow. **a** From the collected images, live and dead *B. mucilaginous* and dust were visually identified (as indicated by colored crosses) and the tagged populations were gated on the original plot. **b** Images of dead *B. mucilaginous* in the fermented liquid. **c** Images of live *B. mucilaginous* in the fermented liquid. Images from within each region were chosen at random. From left to right, bright-field, SYTO 9, and propidium iodide channel images are displayed

A scatter plot comparing fluorescence intensities of SYTO 9 in the green channel and PI in the red channel was generated for the in-focus population. Two segregated regions were identified and gated (Fig. 2a). SYTO 9 stain generally labels all bacteria, including both those with intact and damaged membranes. By contrast, PI penetrates only bacteria with damaged membranes, causing a reduction in SYTO 9 stain fluorescence when both dyes are present. Therefore, when using a combination of SYTO 9 and PI stains, bacteria with intact cell membranes stain fluorescent green, whereas bacteria with damaged membranes stain fluorescent red. Dead bacteria have PI fluorescent signals or both SYTO 9 and PI signals (Fig. 2b). Bacteria with only the SYTO 9 fluorescent signal were considered living (Fig. 2c).

### Single-factor tests

**Fig. 3:**
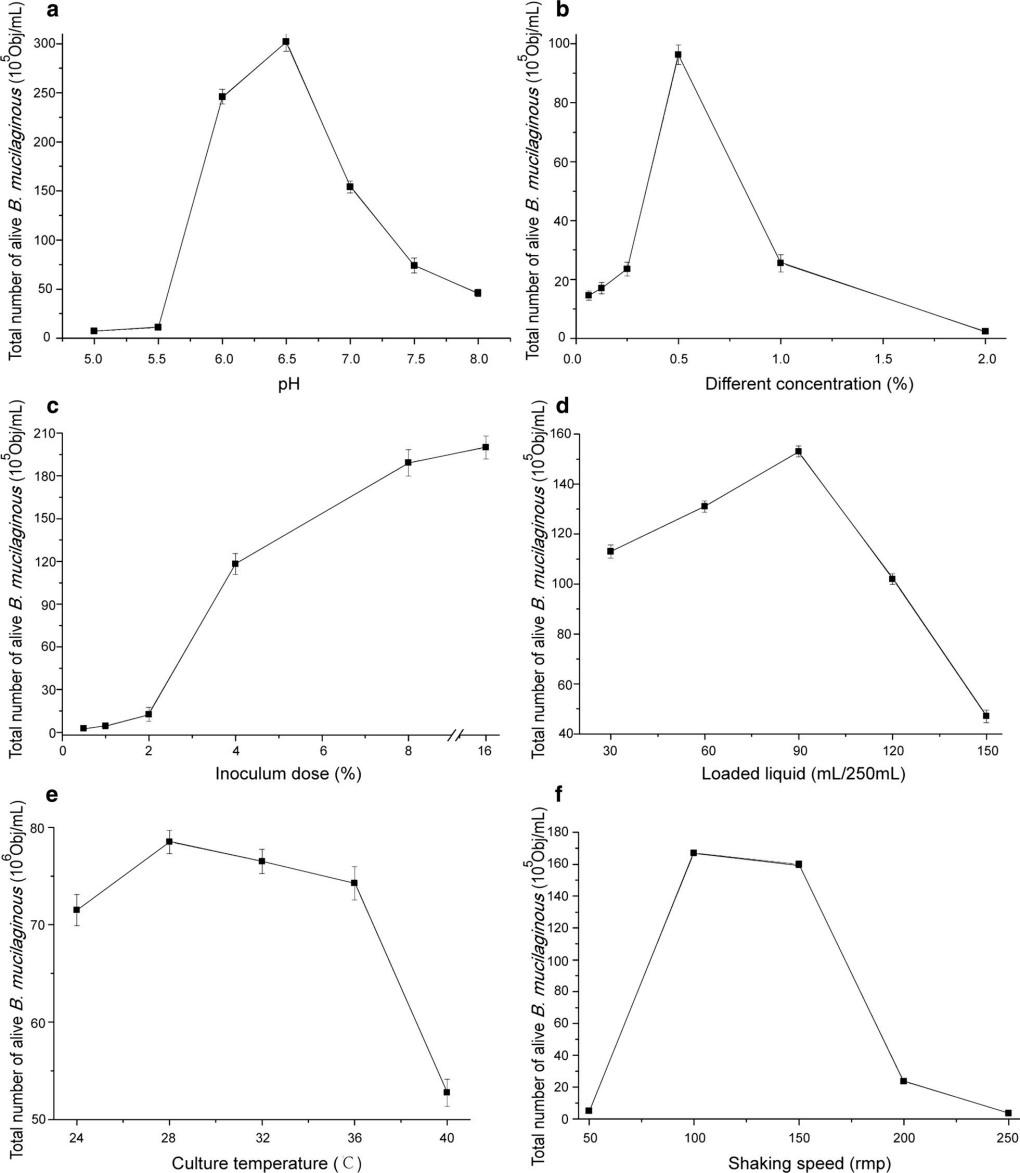
Results of the single-factor test. Effect of (**a**) initial pH, (**b**) concentration, (**c**) inoculum, (**d**) initial volume of loaded liquid, (**e**) culture temperature, and (**f**) shaking speed on total number of live *B. mucilaginous*

As shown in Fig. 3a, as the initial pH value increased, the total number of living bacteria first increased and then decreased. When the pH < 6.5, the total number of viable cells increased significantly. At pH = 6.5, the maximum value was 3.02 × 10^7^ Obj/mL. At pH > 6.5, the total number of living bacteria decreased. When *B. mucilaginous* was in slightly acidic or alkaline conditions, the total number of viable cells decreased, while in neutral or sub-acidic environments, the total number of viable cells was maintained at a high level.

As shown in Fig. 3b, when the solubility of the precooking liquid increased, the total number of live bacteria first increased and then decreased. When the solubility of the precooked solution was 0.5%, the number of viable *B. mucilaginous* was the highest, 9.63 × 10^6^ Obj/mL. Solubility refers to the soluble solid content in the processing wastewater, which directly determines the amount of nutrients supplied to the microorganisms. Fermentation broth with high solubility can provide adequate nutrition to and promote the growth and reproduction of microorganisms. However, when the solubility is too high, the osmotic pressure will increase to levels inhibitory to bacterial growth. Precooked liquid with a lower solubility could satisfy the growth of *B. mucilaginous*, while a solubility of more than 1% could inhibit the growth of *B. mucilaginous*.

As shown in Fig. 3c, as the inoculation volume increased, the total number of living bacteria increased. The inoculation volume was approximately 0.5–2%, and the total number of living bacteria was found to increase rapidly. However, the total amount was low. When the inoculation volume was at approximately 2–8%, the total number of living bacteria was found to steadily increase, and the total number was moderate. When the inoculation volume was approximately 8–16%, the number of living bacteria was found to decrease slightly. However, the inoculation volume directly affects the microbial fermentation cycle. If the inoculum volume is too big, the nutrients and oxygen available to the bacteria in the unit volume will be insufficient when the bacteria proliferate, leading to abnormal metabolism by the bacteria and affecting product synthesis. However, when the inoculum is too little, it not only wastes resources, but also increases the duration of the lag period. This prolongs the fermentation period and significantly increases the cost. From the perspective of cost and efficiency, the appropriate inoculation range was determined to be 4–10%.

For shake flask fermentation, the oxygen transmission coefficient has an indirect relationship with the liquid load. However, the concurrent decrease in the amount of medium with the increase in the oxygen transmission coefficient results in exhaustion of the medium as fermentation progresses, affecting the growth of the bacteria. As shown in Fig. 3d, the total number of viable bacteria first increased and then decreased as the amount of liquid increased. When increasing from 30 to 90 mL/250 mL liquid volume, the number of colonies increased in an almost linear manner, peaking at 1.53 × 10^7^ Obj/mL in 90 mL/250 mL. In 150 mL/250 mL, the total number of colonies decreased significantly to even less than the number of colonies present in smallest amount of liquid tested of 30 mL/250 mL.

Mycelial cell growth involves a series of enzymatic reactions. Temperatures that are either too high or low inhibit the activity of certain enzymes in these cells, which can adversely affect the growth of the cells and product synthesis, as well as cause changes in morphology, metabolism, toxicity against microorganisms, and even lead to cell death. Meanwhile, a suitable temperature can stimulate growth. As shown in Fig. 3e, the total number of living bacteria increased first and then decreased with an increase in temperature. Between 24 and 28 °C, the total number of viable bacteria increased as the temperature increased, but decreased rapidly as temperature increased higher than 28 °C. In particular, when the temperature was 40 °C, the population of viable bacteria was even smaller than at 24 °C. The temperature range suitable for *B. mucilaginous* growth was determined to be 24–36 °C.

There is a positive correlation between shaker speed and the amount of dissolved oxygen, where the latter can also reflect bacterial growth. There is not a high enough amount of dissolved oxygen, as well as uneven mixing of the substances, in the fermentation system when shaking is too slow. The large amount of organic acids produced during the growth of an aerobic decomposition-consuming strain cannot be fully utilized, which greatly reduces the bacterial yield. However, when the shaking speed is too high, the amount of dissolved oxygen increases notably and results in the production of a large number of metabolites, which can also affect bacterial growth. As shown in Fig. 3f, the total amount of viable bacteria first increased and then decreased as the shaker speed increased. The viable bacteria population was largest, 1.67 × 10^7^ Obj/mL, at 100 rpm.

### Three main factors affecting the total number of live *B. mucilaginous* based on Plackett–Burman design

The Plackett–Burman experimental design based on the single-factor experimental results and analysis of variance results is presented in Table 3. The model had a p = 0.0032 < 0.01, indicating this model is extremely significant. Based on the P values of the 6 factors assessed, the factors affecting the total number of *B. mucilaginous* in order of influence were E > D>B > F>C > A. The factors that had a significant impact were E, D, B, and F, i.e., shaking speed, temperature, pH, and liquid volume, respectively. Therefore, shaking speed, temperature, and pH were assessed as key factors in the next experiment.

**Table 3:** Analysis of variance in Plackett–Burman

Source	Sum of squares	*df*	Mean squares	F value	*p* value prob > F	Coefficient estimate	Importance
Model	3.737E	6	62,280.99	17.60	0.0032		
A	623.52	1	623.52	0.18	0.6921	7.21	6
B	64,167.19	1	64,167.19	18.13	0.0080	73.13	3
C	1692.19	1	1692.19	0.48	0.5201	− 11.88	5
D	82,751.02	1	82,751.02	23.38	0.0047	83.04	2
E	1.817E+005	1	1.817E+005	51.33	0.0008	− 123.04	1
F	42,781.02	1	42,781.02	12.09	0.0177	59.71	4
Residual	17,695.27	5	3539.05				
Cor total	3.914E+005	11					

### Steepest ascent design

According to the positive and negative effects of the Coefficient Estimate of E, D, and B three factors depicted in Table 3, culture temperature and the initial pH exhibited positive effects and the shaking speed exhibited a negative effect. The results of the steepest ascent design are shown in Table 4. As the culture temperature and pH gradually increased, the shaking speed was found to gradually decrease, the total number of *B. mucilaginous* first increased and then decreased. The maximum total number of viable *B. mucilaginous* was reached when the rotational speed was 200 rpm, temperature was 28 °C, and pH was 6.5. Therefore, the next step of the response surface experiment was designed using level number 2 as the central value in Table 4.

**Table 4:** Experimental design of steepest ascent and corresponding results

Run	Shaking speed (rmp)	Culture temperature (°C)	pH	Total viable *B. mucilaginous* (10^7^ Obj/mL)
1	250	24	6.0	1.06 ± 0.01
2	200	28	6.5	2.17 ± 0.02
3	150	32	7.0	1.92 ± 0.03
4	100	36	7.5	1.05 ± 0.01
5	50	40	8.0	0.62 ± 0.02

### Response surface experimental design and analysis of results

After the optimal range for the three important factors was determined, response surface analysis was carried out at a shaking speed (*X*_1_) of 200 rpm, temperature (*X*_2_) of 28 °C, and pH (*X*_3_) of 6.5. The level of each variable is shown in Table 5. The total number of viable *B. mucilaginous* was considered the response value (Y) and Design-Expert 8.0 software was used. The two regression model of the total number of *B. mucilaginous* and each factor influencing *B. mucilaginous* growth were as follows:

$$\begin{aligned} {\text{Y}}\, & = \, 1 2 5.00\, - \, 8. 8 1X_{ 1} \, + \, 20. 8 1X_{ 2} \, + \, 2. 7 5X_{ 3} \, - \, 1 1.00X_{ 1} X_{ 2} \, - \, 1 2. 8 8X_{ 1} X_{ 3} \\ & - \, 1 1. 3 7X_{ 2} X_{ 3} \, - \, 60. 7 5X_{ 1}^{ 2} \, - \, 3 5. 50X_{ 2}^{ 2} \, - \, 4 4. 3 8X_{ 3}^{ 2} . \\ \end{aligned}$$

**Table 5:** Box–Behnken design

Run	*X*_1_ [shaking speed (rmp)]	*X*_2_ [culture temperature (°C)]	*X*_3_ (pH)	Total viable *B. mucilaginous* (10^8^ Obj/mL)
1	150	28	7.0	0.44 ± 0.01
2	200	28	6.5	1.15 ± 0.02
3	200	28	6.5	1.16 ± 0.03
4	200	32	6.0	0.77 ± 0.02
5	250	32	6.5	0.27 ± 0.01
6	250	28	6.0	0.21 ± 0.01
7	200	24	6.0	0.09 ± 0.00
8	150	32	6.5	0.68 ± 0.01
9	200	32	7.0	0.58 ± 0.01
10	200	28	6.5	1.18 ± 0.02
11	150	28	6.0	0.11 ± 0.01
12	250	28	7.0	0.03 ± 0.00
13	250	24	6.5	0.11 ± 0.00
14	200	24	7.0	0.35 ± 0.01
15	200	28	6.5	1.20 ± 0.02
16	200	28	6.5	1.10 ± 0.02
17	150	24	6.5	0.09 ± 0.00

The results of variance analysis of the regression model are shown in Tables 6. The regression model had a p < 0.0001, revealing the regression equation used to describe the relationship between every factor and response value yielded a very significant linear relationship between the dependent variable and each independent variable. Overall, the experimental method was reliable. The “Lack of Fit F-value” of 1.03 implies the Lack of Fit is not significant relative to the pure error. There is a 46.83% chance that a “Lack of Fit F-value” could occur due to noise. The model displayed no loss of imitation phenomenon, indicating no abnormalities in the data, more items did not have to be introduced, and the model was appropriate. The parameters *X*_1_, *X*_2_, *X*_1_*X*_2_, *X*_1_*X*_3_, *X*_2_ *X*_3_, *X*_1_^2^, *X*_2_^2^, and *X*_3_^2^ were also significant (p < 0.05), revealing the three factors of speed, temperature, and pH significantly influenced the model. The predicted R^2^ = 0.9756 can also reasonably explain the change in the positive determination coefficient ${\text{R}}_{\text{Adj}}^{2}$ = 0.9929 as there was a good fit between the measured and predicted total number of viable *B. mucilaginous* and it can be used for the theoretical prediction of *B. mucilaginous* fermentation.

**Table 6:** Analysis regression and variance results

Source	Sum of squares	*df*	Mean squares	F value	p-value prob > F	Significance
Model	32,355.03	9	3595.00	249.81	< 0.0001	**
*X*_1_	621.28	1	621.28	43.17	0.0003	**
*X*_2_	3465.28	1	3465.28	240.79	< 0.0001	**
*X*_3_	60.50	1	60.50	4.20	0.0795	
*X*_1_ *·X*_2_	484.00	1	484.00	33.63	0.0007	**
*X*_1_ *·X*_3_	663.06	1	663.06	46.07	0.0003	**
*X*_2_ *·X*_3_	517.56	1	517.56	35.96	0.0005	**
*X*_1_^2^	13,275.04	1	13,275.04	922.45	< 0.0001	**
*X*_2_^2^	4020.25	1	4020.25	279.36	< 0.0001	**
*X*_3_^2^	6661.27	1	6661.27	462.87	< 0.0001	**
Residual	100.74	7	14.39			
Lack of fit	43.94	3	14.65	1.03	0.4683	
Pure error	56.80	4	14.20			
Cor total	32,455.76	16				
R^2^ = 0.9969, ${\text{R}}_{\text{Adj}}^{2}$ = 0.9929, ${\text{R}}_{\text{Pred}}^{2}$ = 0.9756						

* Significant, ** very significant

**Fig. 4:**
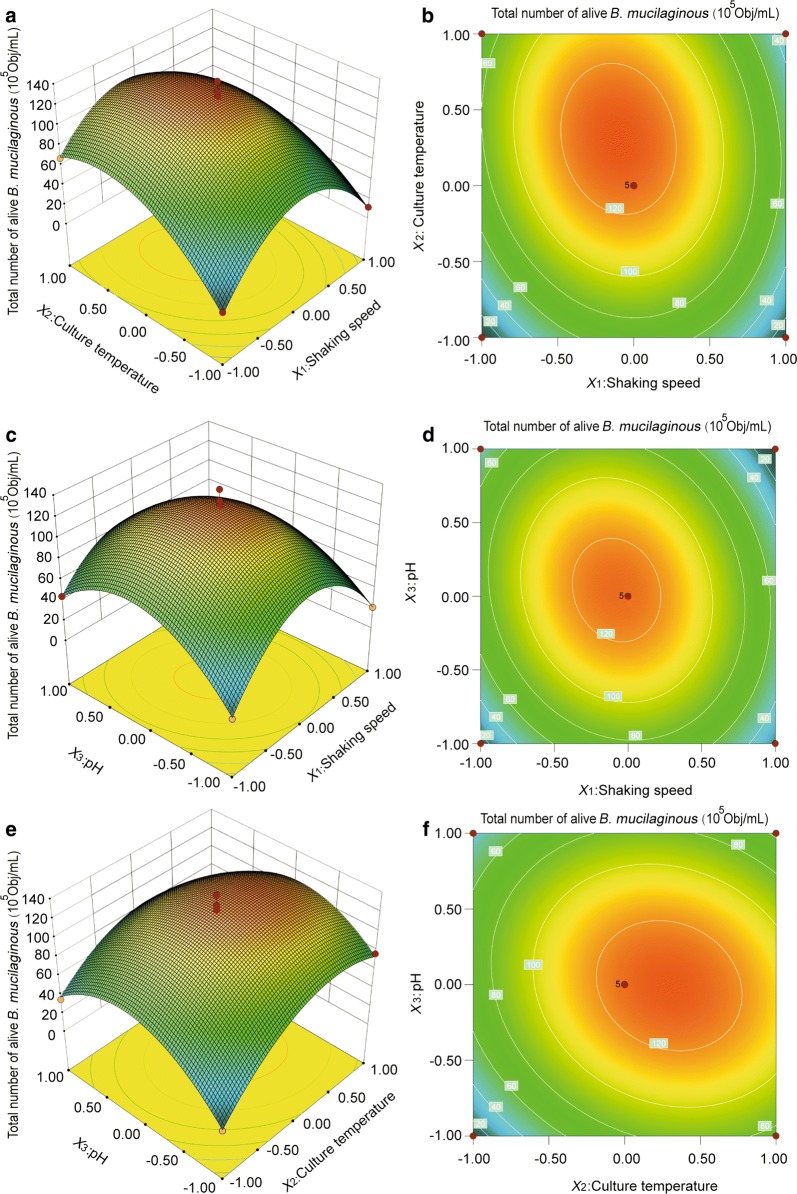
The effect of cross-interaction among shaking speed, culture temperature and pH on total number of alive *B. mucilaginous.* **a** Response surface plot of effects of interaction between shaking speed and culture temperature on total number of alive *B. mucilaginous*; **b** contour line of effects of interaction between shaking speed and culture temperature on total number of alive *B. mucilaginous*; **c** response surface plot of effects of interaction between shaking speed and pH on total number of alive *B. mucilaginous*; **d** contour line of effects of interaction between shaking speed and pH on total number of alive *B. mucilaginous*; **e** response surface plot of effects of interaction between culture temperature and pH on total number of alive *B. mucilaginous*; **f** contour line of effects of interaction between culture temperature and pH on total number of alive *B. mucilaginous*

An analysis chart was generated based on the regression equation to investigate the shape of the response surface. The response surface contour maps for each factor are shown in Fig. 4.

As shown in Fig. 4, the profiles of the response surfaces between speed and temperature, speed and pH, and temperature and pH are all convex with an open downward direction, indicating a high total number of viable *B. mucilaginous*. The contour centers of the three response surfaces are located within the set range, indicating optimal design conditions exist within the designed level of factors.

Analysis of variance revealed the interaction between shaking speed and culture temperature was significant (p < 0.05). As seen in Fig. 4a, the response surface is steep, indicating the obvious influence of speed and culture temperature on the total number of viable bacteria. In addition, the contour line in Fig. 4b is oval and the interaction between shaking speed and temperature was significant. When the pH was 6.5 (pH test level is 0), the total number of living bacteria first gradually increased and then decreased as shaking speed and the culture temperature increased. When the pH was 6.5, shaking speed was 200 rmp, culture temperature was 28 °C, the total number of living bacteria reached the actual maximum closing to the design points.

The response surface in Fig. 4c is steep, indicating the obvious influence of shaking speed and pH on the total number of living bacteria. In addition, the contour line in Fig. 4d is oval and the interaction between speed and pH was significant. Under a constant temperature, the total number of living bacteria first increased and then decreased as the speed and pH increased and the vertex of the surface is the maximum amount of total living bacteria.

The response surface in Fig. 4e is steep, indicating the notable influence of temperature and pH on the total number of living bacteria. In addition, the contour line in Fig. 4f is oval and the interaction between temperature and pH was significant. Under a constant shaking speed (the level is 0), the total number of living bacteria first increased and then decreased as the temperature and inoculum volume increased and the vertex of the surface indicates the maximum amount of total living bacteria.

### Verification test

Based on the above analysis using Design-Expert 8.0 software to optimize the fermentation conditions, the optimal fermentation conditions were a shaking speed of 194.34 rpm, temperature of 29.42 °C, and pH of 6.50, which was predicted to yield a total of 1.20 × 10^8^ Obj/mL viable *B. mucilaginous*. After these values were rounded, each experiment was performed 3 times. The fermentation conditions used for *B. mucilaginous* were a shaking speed of 194 rpm, culture temperature of 29 °C, pH of 6.5, liquid volume of 90 mL/250 mL, inoculums dose of 8%, and pre-cooking liquid solubility of 0.5%. After 48 h, the total number of viable *B. mucilaginous* was 2.16 ± 0.03 × 10^8^ Obj/mL, reaching the requirements of agricultural microbial inoculants, which showed the experimental results were in good agreement with the model.

## Discussion

In the present study, Plackett–Burman was combined with Box–Behnken experimental design to optimize the culture conditions of *B. mucilaginous*. Multispectral imaging flow cytometry was applied to quantify the total number of viable *B. mucilaginous* in fermentation broth. This study aimed to quickly and conveniently establish the optimal culture conditions for *B. mucilaginous*. First, an important statistical technique, Plackett–Burman experimental design with a relatively small number of experiments is a two-level design method based on the principle of incompletely balanced blocks, generally applied to screen the significant variables from a multivariable system, and to provide a foundation for further optimization (He et al. 2014). It can filter out factors that significantly influence experimental results with the fewest experiments and facilitate making experimental results more scientifically. Therefore, it is generally used in media or conditions of fermentation (Atli et al. 2013; Tian et al. 2014; Mohan et al. 2016), methods or technology of extraction (Chen et al. 2015b, 2017b), and so on. In this study, three significant factors including shaking speed, culturing temperature, and initial pH were filtered out with Plackett–Burman. Second, Box–Behnken experimental design is less frequently, the cycle is short, and the regression equation obtained is highly precise. It is also an effective method to study interactions between several factors, which can be used to reduce development costs, optimize processing conditions, improve product quality, and solve practical production problems (Khoshayand et al. 2011; Liang et al. 2011). Third, SYTO 9 and PI were used for double staining of viable and dead cells. With an appropriate mixture of SYTO 9 and PI stains, bacteria with intact cell membranes stain fluorescent green, scored as viable ones; and bacteria with injured membranes stain fluorescent red, scored as non-viable ones (Leuko et al. 2004; Hu et al. 2017). And the fluorescent staining of bacterial cells was quantified with multispectral imaging flow cytometry by fluorescent signals and microscopic image (Berney et al. 2007; Jenner et al. 2016). Therefore, the *B. mucilaginous* viable and dead cells and particles in the fermented liquid were quickly and accurately identified and quantified by flow cytometry in a statistically sound manner.

Due to the short storage period of fresh *A. bisporus*, the main form of international trade of this product is canned processed products. Tank processing must be promptly used to precook fresh mushrooms. The weight of the cooked mushrooms after precooking is 35–40% lower than that of fresh mushrooms and this weight is lost as industrial *A. bisporus* wastewater (Huang et al. 2016). In this wastewater, there are a lot of nutrients available that are suitable for microorganism growth (Huang et al. 2016; Lin et al. 2016). For example, this wastewater is suitable to support *B. mucilaginous* growth when it was an approximately 0.25% solubility, which is lower than that was used as culture medium of *Anoectochilus roxburghii* (Zhan et al. 2017), indicating *A. bisporus* processing wastewater is a good natural medium.

The growth and energy metabolism of microbes are affected by their environmental pH. Since the phases of a bioprocess are dynamic and are the consequences of directed functioning of the bioreaction network interacting strongly with the environment of the cell, the influence of pH on the overall bioreaction, are indeed important (Wang et al. 2011). In this study, *B. mucilaginous* had a higher growth state in neutral or slightly acidic environments (pH of medium was from 6.0 to 7.5), which significantly affected the total number of viable bacteria in the fermentation broth. And more, *B. mucilaginous* fermentation broth was prepared to be neutral or slightly acidic to be used directly for the preparation of microbial fertilizer and reducing costs.

Temperature influences microbial life mainly by affecting the mobility of the microbial cell membrane and activity of biological macromolecules (Baweja et al. 2016; Taniguchi et al. 2017). As temperature increases, the rates of intracellular enzymatic reactions increase, resulting in an increase in cell metabolism and growth. However, once the temperature becomes too high, the bioactive substances become denatured, resulting in decreased cell functions and even death (Kunze et al. 2014). In accordance with this, the total number of viable *B. mucilaginous* first increased and then decreased as the temperature increased. The most suitable temperature range was 24–32 °C with an optimal incubation temperature of 26 °C.

The effect of shaking speed, incubation temperature, and pH and interactions between these factors all had a significant effect on the total number of viable *B. mucilaginous*. After optimization of the response surface, the optimum conditions for the fermentation of *B. mucilaginous* using *A. bisporus* wastewater were determined to be a rotational speed of 194 rpm, solubility of 0.5%, culture temperature of 26 °C, initial pH of 6.5, inoculum of 8%, culture time of 48 h, and amount of liquid loaded of 90 mL/250 mL. Under these conditions, the total number of living bacteria can reach 2.16 ± 0.02 × 10^8^ Obj/mL.

## Abbreviations

*A. bisporus*: *Agaricus bisporus (Lange) Sing*
*B. mucilaginous*: *Bacillus mucilaginous*
PI: propidium iodide
PBS: phosphate buffer solution

## References

[CR1] Atli B, Yama M, Yildiz Z (2013). Optimization of submerged fermentation conditions for lovastatin production by the culinary-medicinal oyster mushroom, Pleurotus ostreatus (Higher Basidiomycetes). Int J Med Mushrooms.

[CR2] Baweja M, Nain L, Kawarabayasi Y, Shukla P (2016). Current technological improvements in enzymes toward their biotechnological applications. Front Microbiol.

[CR3] Berney M, Hammes F, Bosshard F, Weilenmann HU, Egli T (2007). Assessment and interpretation of bacterial viability by using the LIVE/DEAD BacLight kit in combination with flow cytometry. Appl Environ Microbiol.

[CR4] Calvert Meredith EK, Lannigan Joanne A, Pemberton Lucy F (2008). Optimization of yeast cell cycle analysis and morphological characterization by multispectral imaging flow cytometry. Cytometry A.

[CR5] Chen H, Niu J, Qin T, Ma Q, Wang L, Shu G (2015). Optimization of the medium for *Lactobacillus acidophilus* by Plackett–Burman and steepest ascent experiment. Acta Sci Pol Technol Aliment.

[CR6] Chen J, Sun C, Han L, Lin X, Wang L, Shen M, Yu F (2015). Extraction of crude polysaccharides from *Duchesnea indica* (Andrews) Focke: optimization by response surface methodology. Biosci Biotechnol Biochem.

[CR7] Chen H, Huang J, Shi XY, Li YC, Yu L (2017). Effects of six substances on the growth and freeze-drying of *Lactobacillus delbrueckii* subsp. *bulgaricus*. Acta Sci Pol Technol Aliment.

[CR8] Chen F, Zhang Q, Liu J, Gu H, Yang L (2017). An efficient approach for the extraction of orientin and vitexin from *Trollius chinensis* flowers using ultrasonic circulating technique. Ultrason Sonochem.

[CR9] Cui FJ, Zhao LM (2012). Optimization of xylanase production from *Penicillium sp*.WX-Z1 by a two-step statistical strategy: Plackett–Burman and Box–Behnken experimental design. Int J Mol Sci.

[CR10] Dalton H, Postgate JR (1969). Growth and physiology of *Azotobacter chroococcum* in continuous culture. J Gen Microbiol.

[CR11] Gao XJ, Yan PS, Liu X, Wang JB, Yu JJ (2016). Optimization of cultural conditions for antioxidant exopolysaccharides from *Xerocomus badius* grown in shrimp byproduct. Biomed Res Int.

[CR12] Glick BR (2012). Plant growth promoting bacteria: mechanisms and applications. Scientifica.

[CR13] He Y, Xu J, Wang S, Zhou G, Liu J (2014). Optimization of medium components for production of chitin deacetylase by *Bacillus amyloliquefaciens* Z7, using response surface methodology. Biotechnol Biotechnol Equip.

[CR14] Hu W, Murata K, Zhang D (2017). Applicability of LIVE/DEAD BacLight stain with glutaraldehyde fixation for the measurement of bacterial abundance and viability in rainwater. J Environ Sci (China).

[CR15] Huang JF, Ou YX, Yew TW, Liu JN, Leng B, Lin ZC, Su Y, Zhuang YH, Lin JF, Li XM, Xue Y, Pan YT (2016). Hepatoprotective effects of polysaccharide isolated from *Agaricus bisporus* industrial wastewater against CCl(4)-induced hepatic injury in mice. Int J Biol Macromol.

[CR16] Javadi Nobandegani MB, Saud HM, Yun WM (2015). Phylogenetic relationship of phosphate solubilizing bacteria according to 16S rRNA genes. Biomed Res Int.

[CR17] Jenner D, Ducker C, Clark G, Prior J, Rowland CA (2016). Using multispectral imaging flow cytometry to assess an in vitro intracellular *Burkholderia thailandensis* infection model. Cytometry A.

[CR18] Kang C, Wen TC, Kang JC, Meng ZB, Li GR, Hyde KD (2014). Optimization of large-scale culture conditions for the production of cordycepin with *Cordyceps militaris* by liquid static culture. Sci World J.

[CR19] Khalid M, Hassani D, Bilal M, Asad F, Huang D (2017). Influence of bio-fertilizer containing beneficial fungi and rhizospheric bacteria on health promoting compounds and antioxidant activity of *Spinacia oleracea* L. Botanical Studies.

[CR20] Khoshayand F, Goodarzi S, Shahverdi AR, Khoshayand MR (2011). Optimization of culture conditions for fermentation of soymilk using *Lactobacillus casei* by response surface methodology. Probiotics Antimicrob Proteins.

[CR21] Koroney AS, Plasson C, Pawlak B, Sidikou R, Driouich A, Menu-Bouaouiche L, Vicré-Gibouin M (2016). Root exudate of *Solanum tuberosum* is enriched in galactose-containing molecules and impacts the growth of *Pectobacterium atrosepticum*. Ann Bot.

[CR22] Kuan KB, Othman R, Abdul Rahim K, Shamsuddin ZH (2016). Plant growth promoting rhizobacteria inoculation to enhance vegetative growth, nitrogen fixation and nitrogen remobilisation of maize under greenhouse conditions. PLoS ONE.

[CR23] Kunze M, Lattermann C, Diederichs S, Kroutil W, Büchs J (2014). Minireactor-based high-throughput temperature profiling for the optimization of microbial and enzymatic processes. J Bio Eng.

[CR24] Leuko S, Legat A, Fendrihan S, Stan-Lotter H (2004). Evaluation of the LIVE/DEAD BacLight kit for detection of extremophilic archaea and visualization of microorganisms in environmental hypersaline samples. Appl Environ Microbiol.

[CR25] Liang JD, Han YF, Zhang JW, Du W, Liang ZQ, Li ZZ (2011). Optimal culture conditions for keratinase production by a novel thermophilic *Myceliophthora thermophila* strain GZUIFR-H49-1. J Appl Microbiol.

[CR26] Lin JM, Huang JF, Chen JY, Lin ZC, Ou YX, Yao LY, Zhang XF, Kang QJ, Pan YT (2016). Low cytotoxic d-mannitol isolated from the industrial wastewater of *Agaricus bisporus*. J Food Nutr Res.

[CR27] Mohan N, Balakrishnan R, Sivaprakasam S (2016). Optimization and effect of dairy industrial waste as media components in the production of hyaluronic acid by *Streptococcus thermophilus*. Prep Biochem Biotechnol.

[CR28] Ren XY, He L, Cheng JW, Chang JM (2014). Optimization of the solid-state fermentation and properties of a polysaccharide from *Paecilomyces cicadae (Miquel) Samson* and its antioxidant activities *in vitro*. PLoS ONE.

[CR29] Rojas A, Holguin G, Glick BR, Bashan Y (2001). Synergism between *Phyllobacterium* sp. (N_2_-fixer) and *Bacillus licheniformis* (P-solubilizer), both from a semiarid mangrove rhizosphere. FEMS Microbiol Ecol.

[CR30] Schütz L, Gattinger A, Meier M, Müller A, Boller T, Mäder P, Mathimaran N (2017). Improving crop yield and nutrient use efficiency via biofertilization—a global meta-analysis. Front Plant Sci.

[CR31] Taniguchi H, Sungwallek S, Chotchuang P, Okano K, Honda K (2017). A key enzyme of the NAD(+) salvage pathway in *Thermus thermophilus*: characterization of nicotinamidase and the impact of its gene deletion at high temperatures. J Bacteriol.

[CR32] Tian Y, Fan Y, Zhao X, Zhang J, Yang L, Liu J (2014). Optimization of fermentation medium for acetoin production by *Bacillus subtilis* SF4-3 using statistical methods. Prep Biochem Biotechnol.

[CR33] Wang Y, Fang X, Cheng Y, Zhang X (2011). Manipulation of pH shift to enhance the growth and antibiotic activity of *Xenorhabdus nematophila*. J Biomed Biotechnol.

[CR34] Wang DD, Zhuo CY, Zhu XS, Li TB (2016). Optimization of fermentation condition of *Pichia pastoris* for xylanase production by response analysis. Sci Tec Food Ind.

[CR35] Zhan XR, Huang JF, Chen JM, Pan YT (2017). Optimization of tissue culture medium of *Anoectochilus roxburghii* using liquid of the mushroom precooking process (LMPP). J Minnan Normol Univ.

[CR36] Zhang WB, He XL, Liu HN, Guo HY, Ren FZ, Wen PC (2013). Statistical optimization of culture conditions for milk-clotting enzyme production by *Bacillus amyloliquefaciens* using wheat bran-an agro-industry waste. Indian J Microbiol.

